# O-demethyl galantamine alters protein expression in cerebellum of 5xFAD mice

**DOI:** 10.55730/1300-0152.2692

**Published:** 2024-05-28

**Authors:** İrem KIRIŞ, Merve KARAYEL BAŞAR, Büşra GÜREL, Tomasz MROCZEK, Ahmet Tarık BAYKAL

**Affiliations:** 1 Department of Biochemistry and Molecular Biology, Institute of Health Sciences, Acıbadem Mehmet Ali Aydınlar University, İstanbul, Turkiye; 2 Faculty of Engineering and Natural Sciences, Sabancı University, İstanbul, Turkiye; 3 Nanotechnology Research and Application Center, SUNUM, Sabancı University, İstanbul, Turkiye; 4 Chair and Department of Pharmacognosy, Medical University of Lublin, Lublin, Poland; 5 Department of Medical Biochemistry, Faculty of Medicine, Acıbadem Mehmet Ali Aydınlar University, İstanbul, Turkiye

**Keywords:** Alzheimer’s disease, 5xFAD, o-demethyl galantamine, sanguinine, neurodegeneration, label-free proteomics

## Abstract

**Background/aim:**

Alzheimer’s disease (AD), one of the most common health issues, is characterized by memory loss, severe behavioral disorders, and eventually death. Despite many studies, there are still no drugs that can treat AD or stop it from progressing. Previous in vitro tests showed that O-demethyl galantamine (ODG) might have therapeutic potential owing to its 10 times higher acetylcholinesterase inhibitory activity than galantamine (GAL).

**Materials and methods:**

We aimed to assess the effect of ODG at the molecular level in a 12-month-old 5xFAD Alzheimer’s mouse model. To this end, following the administrations of ODG and GAL (used as a positive control), protein alterations were investigated in the cortex, hippocampus, and cerebellum regions of the brain. Surprisingly, GAL altered proteins prominently in the cortex, while ODG exclusively exerted its effect on the cerebellum.

**Results:**

GNB1, GNB2, NDUFS6, PAK2, and RhoA proteins were identified as the top 5 hub proteins in the cerebellum of ODG-treated mice. Reregulation of these proteins through Ras signaling and retrograde endocannabinoid signaling pathways, which were found to be enriched, might contribute to reversing AD-induced molecular changes.

**Conclusion:**

We suggest that, since it targets specifically the cerebellum, ODG may be further evaluated for combination therapies for AD.

## 1. Introduction

Alzheimer’s disease (AD) is a prevalent neurodegenerative disorder characterized by amyloid-β (Aβ) plaques and neurofibrillary tangles (NFTs). Pathologic signs of AD include atrophy, neuroinflammation, and synaptic failure. Memory loss, confusion about time and location, speaking and writing issues, mood and personality changes, and other clinical indications of AD effectively disrupt daily activities, and patients finally become bedridden (Yamada and Nabeshima, 2000). In 2021, AD was the seventh leading cause of death, and dementia is now considered a global epidemic, affecting 55 million people, with projections reaching 78 million by 2030. Along with the number of patients, the social and economic burden is increasing year after year. Efforts to discover and develop viable treatments for AD have continued worldwide for many years. However, there are currently no drugs available to treat or halt the progression of AD (Gauthier et al., 2021). FDA-approved drugs only provide temporary relief and/or slow the progression of the illness from mild to moderate stages.

**Figure 1:**
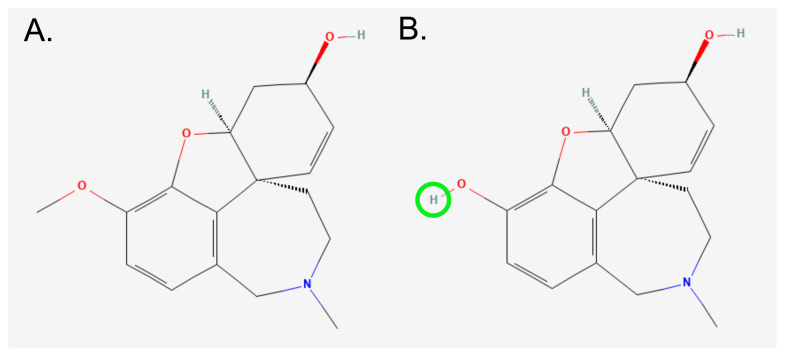
Chemical structure of (A) Galantamine (GAL) and (B) O-demethyl galantamine (ODG). ODG is formed from GAL by the removal of the methyl group, from the ring (highlighted as green). (GAL and ODG’s PubChem CID: 9651 and 443722, respectively).

O-demethyl galantamine (ODG), also called sanguinine, has been studied using TLC-bioautography and HPLC/HR-MS (Mroczek, 2009, 2016; Mroczek et al., 2020). It exhibited prominent acetylcholinesterase inhibitory activity, approximately 10 times higher than galantamine (GAL), which has been used as an FDA-approved AD drug (Bores et al., 1996; López et al., 2002). This might be caused by the extra hydroxyl group in the ring (Figure 1) (López et al., 2002). Even though it is not often encountered in plant materials, based on our studies, small amounts (<0.01 %) have been found in *Narcissus cv. Hawera* bulbs (Mroczek, 2016; Mroczek et al., 2020). Our promising results in in vitro testing led us to investigate ODG’s effects at the molecular level on mice with AD.

To do so, we used a 5xFAD mouse with 5 Familial AD mutations, a well-known transgenic AD model. Most pathologic abnormalities seen in human AD, such as Aβ-plaques, gliosis, synaptic degeneration, neuronal loss, and progressive cognitive impairments, are manifested in these transgenic mice (Forner et al., 2021). Furthermore, studies pointed out that a large number of differentially expressed genes in 12-month-old 5xFAD are strongly connected to AD patients, particularly to pathways associated with cognitive impairment. Although 5xFAD mice did not show the tau pathology seen in humans, they did show a variety of biochemical and behavioral characteristics associated with AD. Therefore, based on the available data, 12-month-old 5xFAD mice are an appropriate model for studying the effects of a potential therapeutic (Oakley et al., 2006; Bouter et al., 2014; Gurel et al., 2019). The underlying mechanism of ODG in the brain’s cortex, hippocampus, and cerebellum regions was investigated via proteomic analyses. This powerful and widely used technique identifies changes in protein expression, aiding in the understanding of how protein levels vary under various chemical, physical, and biological conditions.

In summary, this study aimed to reveal the working mechanism of ODG in 5xFAD mice through proteomic alterations analyzed via LC-MS/MS.

## 2. Materials and methods

### 2.1. ODG and GAL isolation

GAL and ODG were isolated from bulbs of *Narcissus jonquilla* ‘Pipit’, and *Narcissus cv. ‘Hawera’*, respectively, according to the procedure described in our previous papers, using comprehensive chromatographic techniques like VLC and PLC (Mroczek et al., 2020; Kiris et al., 2021).

### 2.2. Animals and natural compound administration

The 5xFAD transgenic mice (Tg6799, The Jackson Laboratory, Stock no: 034840-JAX) were housed at a constant temperature (22 ± 2 °C) with a 12 h day/12 h night cycle. Food and water were available ad libitum.

GAL and ODG were dissolved in DMSO and diluted with saline solution. The amount of DMSO injected to the mice was kept below 1%. As a positive control, 2 mg/kg of galantamine (GAL, n = 8) was administered. For the treatment groups, 1 mg/kg (low dose of ODG, LODG, n = 6) or 2 mg/kg (high dose of ODG, HODG, n = 6) of ODG was intraperitoneally administered to 12-month-old 5xFAD mice. To eliminate the possible effects of DMSO, 1% DMSO in saline solution was injected to the AD control group (ALZ) (n = 8).

After 7 days of administration, the mice were anesthetized with isoflurane and sacrificed by decapitation. The brains were then removed, quickly sectioned into the cortex, hippocampus, and cerebellum regions, and preserved at −80 °C.

### 2.3. Nano LC-MS/MS analysis

Sample preparation, LC-MS/MS instrument, and data processing parameters were previously reported in detail (Kiris et al., 2022). Briefly, tissues were ground with steel beads, dissolved in UPX (Expedeon) containing protease inhibitor cocktail (Thermo Scientific) using an ultrasonic processor (VialTweeter, HielsScher), and then heated in a thermoshaker at 95 °C for 10 min and centrifuged at 14000 × *g* for 10 min. Samples in each group were pooled into 3 samples. Peptides were prepared using the filter-aided sample preparation (FASP) method. Buffer exchange and alkylation were achieved using 8M urea and 50 mM iodoacetamide (IAA), respectively. The proteins were then kept in trypsin (enzyme-to-substrate ratio: 1:100) overnight at 37 °C. Finally, 200 μg/mL of peptide with 0.1% FA was prepared for LC-MS/MS analysis.

An ACQUITY UPLC M-Class connected to a SYNAPT Xevo G2-XS instrument (Waters) was used for the LC-MS/MS study. Peptides were initially trapped on a trap column (Symmetry C18, 5 μm, 180 μm i.d. × 20 mm) and then separated using gradient elution on an analytic column (CSH C18, 1.7 μm, 75 μm i.d. × 250 mm). A lock mass reference of 100 fmol/uL Glu-1-fibrinopeptide B was utilized. The device was operated in positive ion mode. SONAR, an independent data acquisition mode, was used for MS data gathering, with a 24 Da quadrupole transmission width. All ions in the 50–1950 m/z range were fragmented collectively in the absence of any precursor ion preselection insensitivity mode. Progenesis-QI for proteomics software (V.2.0 Waters) was used to analyze the data by using Mus musculus database (UniProt, 18.01.2022). For low and high energy, peak intensity thresholds were chosen at 60 and 10 counts, respectively. The maximum number of missed cleavages allowed for tryptic digestion was set to 1. Carbamidomethyl modification of cysteine residues (C) was specified as a fixed modification. Additionally, oxidation of methionine residues (M) and deamidation of asparagine (N) and glutamine (Q) residues were considered as variable modifications. A false discovery rate (FDR) of 1% was applied. The total ion intensity normalization was performed. Progenesis QI for proteomics’ statistical program was used to calculate expressional changes, as well as p and q values. Proteins were accepted as differentially expressed (DEP) if only met these criteria: ANOVA p-value < 0.05, q-value < 0.05, unique peptide > 2, and fold change ≥ 1.5.

### 2.4. Bioinformatic analysis

Various software tools were utilized for the analysis and visualization of the LC-MS/MS data, as previously reported (Kiris et al., 2023). The online Clustvis program was used for principal component analysis (PCA) and hierarchical clustering (HC). For PCA, rows were subjected to unit variance scaling, and principal components were calculated using singular value decomposition (SVD) with imputation. Prediction ellipses are designed so that a new observation from the same group will fall inside the ellipse with a probability of 0.95. For HC, rows were centered, and unit variance scaling was applied to rows. Both rows and columns are clustered using Euclidean distance and complete linkage.

The Database for Annotation, Visualization, and Integrated Discovery (DAVID v 6.8) tool was used for GO functional enrichment analysis and Kyoto Encyclopedia of Genes and Genomes (KEGG) pathway analysis. Bubble plots were created by using obtained data on an online platform^1^. Additionally, the final arrangements of figures were prepared using Inkscape software^2^.

Protein-protein interaction analysis was performed with Cytoscape software (3.8.2) with medium stringency. The top 5 hub proteins were identified using the local-based density of maximum neighborhood component (DMNC) method within the CytoHubba plug-in.

## 3. Results

### 3.1. Characterization of compounds

GAL was isolated with a purity higher than 95%. Its chemical structure was established using the LC/HR-MS method. ODG was isolated from *Narcissus cv. Hawera* bulbs with purities higher than 90%. The chemical structure has been confirmed with the HPLC-ESI-QTOF-MS method. The retention times and MS spectral data of both compounds matched those determined for standard compounds. However, only a few milligrams of ODG could be isolated; therefore, it was purchased from American Custom Chemicals (San Diego, USA) for biological tests.

### 3.2. Proteome analysis of the brain regions

Label-free nano LC-MS/MS was used to reveal ODG-driven molecular changes in the Alzheimer’s mouse model brains. Additionally, we analyzed GAL-injected mice brains to compare and contrast with the ODG effects on a protein basis. In ODG groups, 1597, 1653, and 1334 proteins were identified in the cortex, hippocampus, and cerebellum, respectively (Table). GAL only affected the proteins in the cortex with 81 DEPs (Table). However, ODG led to differential alterations only in the cerebellum, with only one DEP detected in the cortex in HODG. All identified proteins and DEPs were presented in detail in Supplementary Table. As shown in Table, 43 proteins were altered in LODG, while 54 proteins were altered in the cerebellum. Since the main focus of this research is ODG, further bioinformatics analyses were carried out on the cerebellum only.

**Figure 2:**
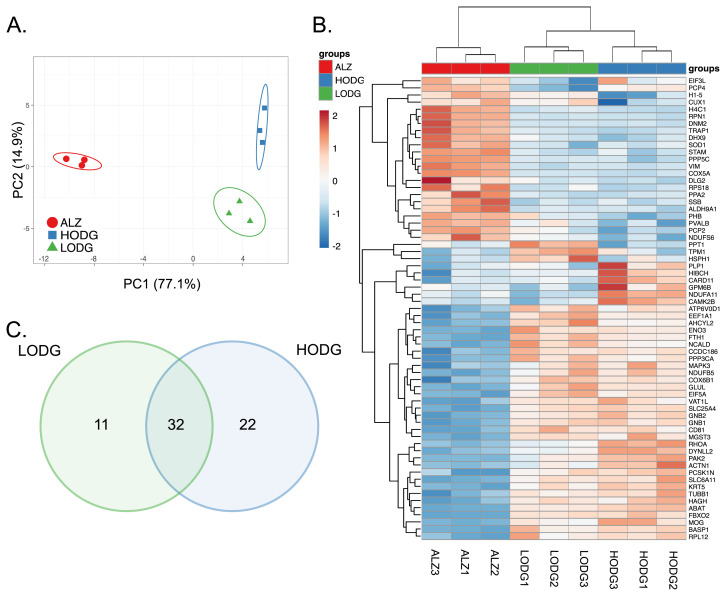
Differential expression of proteins in ODG-administered cerebellum regions. (A) PCA analysis, (B) Heatmap of the DEPs, (C) Venn diagram representing the distribution of DEPs in LODG and HODG compared to the ALZ group.

Firstly, unbiased principle component analysis (PCA) revealed that the proteome of all groups in the cerebellum was greatly altered with PC1 (77.1%) and PC2 (14.9%) (Figure 2A). Additionally, the stringency of the circles, calculated by the Clustvis program, indicated that the results are highly reproducible, with a high probability that a new sample from the group would fall within the same circle. HC also confirmed these results via clustering the samples from the same group together (Figure 2B). The Venn diagram revealed common and specific protein changes in LODG and HODG. It showed that low and high doses of ODG caused highly similar changes in the cerebellum of 5xFAD mice. 32 DEPs were common to both groups, while 11 and 22 DEPs were specific to LODG and HODG, respectively (Figure 2C).

**Figure 3:**
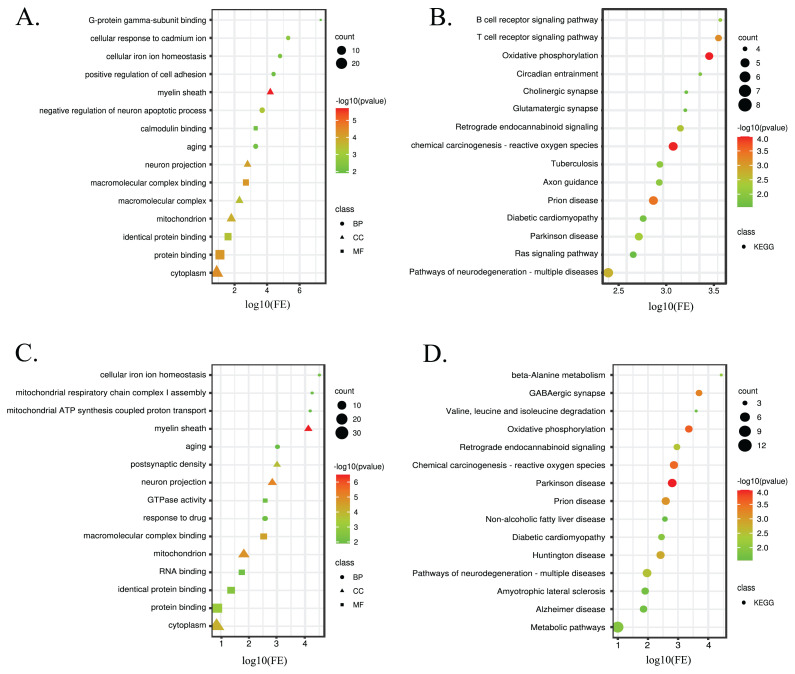
Enrichment analyses of LODG and HODG compared to ALZ are represented in bubble plots. (A) GO analysis and (B) KEGG pathway analysis of DEPs in LODG. (C) GO analysis and (D) KEGG pathway analysis of DEPs in HODG. The GO/KEGG terms are represented on the y-axis. The x-axis showed the fold of enrichment. In the GO analysis plot, circle, triangle, and square represent biological process (BP), cellular component (CC), and molecular function (MF), respectively. The size of the symbol denotes the number of protein-coding genes associated with a given term. The color represents the altered p-value (−log10).

To understand the biological functions of these DEPs, GO enrichment and KEGG pathway analyses were performed, and the results are presented in Figure 3. In comparison to ALZ, DEPS in LODG were enriched in biological processes related to negative regulation of neuron apoptotic process, cellular response to cadmium ion, and positive regulation of cell adhesion. Conversely, the biological processes enriched in DEPs in HODG compared to ALZ were exclusively related to the response to drugs, mitochondrial respiratory chain complex I assembly, and mitochondrial ATP synthesis coupled proton transport. Commonly, LODG and HODG proteins were enriched in cellular iron homeostasis and aging biological processes. It has been known that there is an imbalance in iron homeostasis in the AD brain resulting in cognitive, memory, motor, and other nerve damages (Peng et al., 2021). FTH1 Ferritin heavy chain (FTH1), V-type proton ATPase subunit d 1 (ATP6V0D1), Superoxide dismutase [Cu-Zn] (SOD1) proteins, which were altered in both LODG and HODG, were found to be enriched in this pathway. Aging is one of the most profound risk factors for AD (Liu et al., 2024). In this study, serine/threonine-protein phosphatase 2B catalytic subunit alpha isoform (PPP3CA), mitogen-activated protein kinase 3 (MAPK3), eukaryotic translation initiation factor 5A-1 (EIF5A), and SOD1 proteins that were altered in LODG and 4-aminobutyrate aminotransferase (ABAT), Beta-enolase (ENO3), EIF5A, and SOD1 proteins, which were altered in HODG, were found to be enriched in the aging pathway. Most DEPs in LODG and HODG were found to play a role in binding, namely macromolecular complex binding, protein binding, and identical protein binding. In addition, calmodulin binding and G-protein gamma-subunit binding were observed in LODG, while RNA binding and GTPase activity were seen in HODG in molecular functions. Localization sites of the DEPs in both groups were detected as the myelin sheath, cytoplasm, neuron projection, and mitochondrion.

Additionally, KEGG pathways revealed that DEPs in LODG were enriched in axon guidance, circadian entrainment, cholinergic synapse, glutamatergic synapse, and Ras signaling pathways. HODG proteins were specifically enriched in beta-alanine metabolism, GABAergic synapse, valine, leucine, and isoleucine degradation, nonalcoholic fatty liver disease, Huntington’s disease (HD), amyotrophic lateral sclerosis (ALS), and AD pathways, whereas DEPs in both groups are commonly enriched in pathways of neurodegeneration, such as multiple diseases, chemical carcinogenesis that lead to the formation of reactive oxygen species, prion disease, oxidative phosphorylation (OXPHOS), retrograde endocannabinoid signaling, and Parkinson’s disease (PD). As expected, most of the pathways that DEPs were enriched in had been previously associated with neurodegenerative diseases. However, they were enriched most significantly in the OXPHOS pathway. Dysregulation of OXPHOS leads to decrease in ATP synthesis and elevated ROS. In the long run, this increases the rate of apoptotic cell death in the central nervous system, affecting neurons along with other cell types. The increase in cell death related to the disease results in significant damage to the patients’ brains (Atlante et al., 2022). We have found that NADH dehydrogenase [ubiquinone] 1 alpha subcomplex subunit 11 (NDUFA11), inorganic pyrophosphatase 2 (PPA2), NADH dehydrogenase [ubiquinone] iron-sulfur protein 6 (NDUFS6), V-type proton ATPase subunit d 1 (ATP6V0D1), cytochrome c oxidase subunit 6B1 (COX6B1), Cytochrome c oxidase subunit 5A (COX5A) (altered only in LODG) and NADH dehydrogenase [ubiquinone] 1 beta subcomplex subunit 5 (NDUFB5) (altered only in HODG) proteins, which were differentially expressed in LODG and HODG, were enriched in OXPHOS pathway.

**Figure 4:**
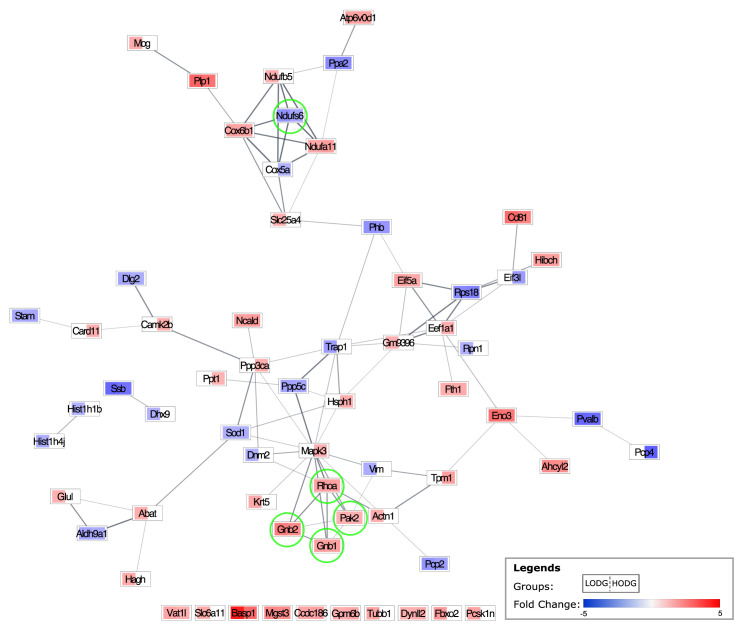
The protein-protein interaction network map of the DEPs in the LODG and HODG groups in comparison to the ALZ group, individually. Each node represents a DEP. The left and right side of the rectangle represents the LODG vs. ALZ, and HODG vs. ALZ, respectively. The upregulation of a DEP is represented in red, and downregulation in blue. The lightness and darkness of the color change gradually due to the fold change (min:−5, max: 5). The white color indicates that protein is not differentially expressed in the given group. The top 5 hub DEPs are indicated with light green circles.

To analyze the interactions between DEPs and identify core regulatory genes, PPI analyses were performed (Athanasios et al., 2017). All DEPs found to be regulated by LODG and/or HODG were subjected to the PPI analysis with STRING. Using Cytoscape, the fold changes were appointed to each protein for respective groups (Figure 4). GNB1, GNB2, NDUFS6, PAK2, and RhoA were detected as the top 5 hub proteins. Surprisingly, they were found to be altered in both LODG and HODG. Moreover, their effects on the expression were in the same direction in both groups. Hence, these proteins might be the key to enlightening the ODG’s working mechanism on AD. Further pathway analysis via KEGG detected that GBB1, GBB2, PAK2, and RhoA proteins are involved in the Ras signaling pathway (Figure S1), while GBB1, GBB2, and NDUFS6 proteins play a role in retrograde endocannabinoid signaling pathway (Figure S2).

### 3.3. Discussion

In previous in vitro studies, ODG has shown greater cholinesterase inhibitor activity compared to GAL (utilized as a positive control in this study due to its structural similarity). However, there is currently no information available regarding its in vivo effects and the underlying mechanism. Therefore, this study aims to evaluate its biological effects and elucidate possible working mechanisms on AD using the powerful proteomics tool LC-MS/MS.

Regional proteomic studies provide more focused results compared to analyzing whole brain tissue, making it easier to extract biologically relevant information. In this study, proteomic analyses were performed in 3 brain regions: the cortex, hippocampus, and cerebellum. We found that GAL affected the expression of proteins in the cortex, while, surprisingly, most of the altered proteins in ODG groups were detected in the cerebellum. Previously, the cerebellum was regarded to be a passive bystander in AD. Recent research has revealed that the cerebellum plays a role in AD by enduring structural, functional, and degenerative changes (Dos Santos et al., 2011; Guo et al., 2016; Gellersen et al., 2021). Cerebellar volume has been linked to cognitive function, memory, and thinking structure (Wassink et al., 1999; Picard et al., 2008; Dos Santos et al., 2011), and it decreases with the severity of dementia (Baldaçara et al., 2011). Two recent genome-wide association studies found that genes associated with intelligence and cognitive function were predominantly expressed in the cerebellum, as well as the cortex (Lam et al., 2017; Savage et al., 2018).

To identify the potential key regulators of the ODG in the AD mouse cerebellum, a PPI network analysis was carried out. The top 5 detected hub proteins (GNB1, GNB2, NDUFS6, PAK2, RhoA) were found to be similarly altered by both low and high dose ODG administration (Figure 4), supporting their involvement in the mechanism of ODG action. To elucidate the mechanism in which they are involved, hub proteins were further analyzed. KEGG analysis revealed that GBB1, GBB2, PAK2, and RhoA proteins are involved in the Ras signaling pathway, whereas GBB1, GBB2, and NDUFS6 proteins play a role in the retrograde endocannabinoid signaling pathway. The relevance of these proteins to AD through the pathways in which they are involved and their reported expression changes in neurodegenerative diseases are briefly discussed below.

The Ras protein superfamily consists of small GTPases that act as molecular switches. They play various critical roles in cellular processes such as neurogenesis, differentiation, gene expression, membrane and protein trafficking, vesicular trafficking, and synaptic plasticity (Sastre et al., 2020). Small GTPases of the Ras family are implicated in neurodegenerative diseases such as AD, PD, and ALS. Ras and Rho families are the most studied families involved in neurodegeneration (Qu et al., 2019).

Transforming protein RhoA (RhoA) belongs to the Rho GTPase family and mediates the formation of stress fibers and focal adhesions. RhoA is involved in several neurodegenerative disorders, including AD, PD, HD, and ALS (Fujita and Yamashita, 2014). Dysregulation of RhoA is thought to contribute to AD pathogenesis by increasing neurite retraction, Aβ aggregation, tau hyperphosphorylation, neuroinflammation, and synaptic damage (Cai et al., 2021; Schmidt et al., 2022). RhoA expression differs with subcellular location; it is decreased in synapses and increased in neurites. RhoA levels are reported to be reduced in the hippocampus of AD patients and AD mice (Huesa et al., 2010). Moreover, Aβ treatment on human neuroblastoma cells led to an increase in RhoA activation and a decrease in Rac1 activation (Petratos et al., 2008; Stankiewicz and Linseman, 2014).

Serine/threonine-protein kinase PAK2 (PAK2), a Rac1 effector, functions in neuronal migration, supports actin formation, and promotes dendritic spine formation (Bokoch 2003; Shin et al., 2009). PAK2 is prominently located in the clusters of cholinergic and monoaminergic and enteric neurons (Zhang et al., 2022). Activation of PAK2 along with other family members is essential to the long-term synaptic plasticity of glutamatergic synapses (Santini et al., 2017). Neurological illnesses linked to PAK2 mutations include autism spectrum disorder, 3q29 microdeletion syndrome, and AD. In in vitro AD models, PAK family proteins have been linked to defective dendritic spine formation (Marlin et al., 2011). The double negative knock-out PAK mice showed memory impairments and PAK2 dysfunction led to autistic-like behaviors, reduced LTP, and decreased synaptic densities, implicating its increase might be beneficial in AD (Wang et al., 2018). Moreover, PAK2 levels were reported to be reduced in the severe AD mice (Nguyen et al., 2008; Civiero and Greggio, 2018).

Guanine nucleotide-binding protein G(I)/G(S)/G(T) subunit beta-1 (GBB1) and guanine nucleotide-binding protein G(I)/G(S)/G(T) subunit beta-2 (GBB2) proteins are β subunits that, together with α and γ subunits, form heterotrimeric G-proteins. G-proteins act as a molecular switch and mediate the signal transduction of G-protein-coupled receptors (GPCRs) (Syrovatkina et al., 2016). GBB1 has been linked to a variety of neurological disorders, including developmental delay, dystonia, growth retardation, and seizures (Petrovski et al., 2016; Hemati et al., 2018). GBB2 also has been associated with neurological diseases such as schizophrenia and neurodevelopmental disorder with hypotonia and dysmorphic facies (Lansdon et al., 2021). GBB1 was found to be decreased in mitochondria of AD mice brains (Martins-de-Souza et al., 2010; Fukuda et al., 2020). GBB2 was reported to be increased in microglial extracellular vesicles in human AD brain tissues (Cohn et al., 2021), whereas it was decreased in entorhinal cortices (Pang et al., 2017).

Endocannabinoids are retrograde messengers that regulate synaptic efficacy and neuronal activity by adjusting the timing of neurotransmitter release from the presynapse (Katona 2006; Ludányi et al., 2011). Even though cannabinoid functions have primarily been seen in the hippocampus, their receptors are mainly located in the cerebellum, brainstem, and microglia (Sánchez et al., 2001; D’Addario et al., 2013). GBB1, GBB2, and NDUFS6 were involved in this pathway; however, since we already discussed GBB1 and GBB2 above, only NDUFS will be mentioned here.

NADH dehydrogenase [ubiquinone] iron-sulfur protein 6 (NDUFS6) is a subunit of the NADH: ubiquinone oxidoreductase (complex I). This complex functions in the transfer of electrons from NADH to the respiratory chain. This protein has been previously reported to be involved in schizophrenia, and Complex I (CI) deficiency, where one of the clinical presentations is neurodegeneration (Martins-De-Souza et al., 2009). However, to our knowledge, there is no publication in the literature regarding expression levels of NDUFS6.

The hub proteins were significantly altered in both high and low doses of ODG, which supports our findings regarding their involvement in the working mechanism of ODG. Specifically, GBB1, GBB2, RhoA, and PAK2 proteins were found to be upregulated, while NDUFS6 was downregulated following ODG administration in the cerebellum. Due to differential expression levels of proteins between different brain regions, and to the best of our knowledge, no study has reported on these specific protein alterations in this particular region. Therefore, we choose not to compare their alterations. However, we speculate that the reregulation of these proteins through retrograde endocannabinoid and Ras signaling pathways might contribute to reversing AD-induced molecular changes.

In conclusion, this is the first in vivo study that evaluates ODG’s potential as a therapeutic agent in AD owing its remarkably higher AChE inhibitory activity than an FDA-approved drug, galantamine. Surprisingly, we revealed that ODG exclusively affected cerebellum proteins whereas galantamine altered cortical proteins. Studies have shown that combination therapies are much more successful in AD than single-target therapies due to the complex nature of the disease (Tanvir Kabir et al., 2020; Uddin et al., 2021). Therefore, we also suggest ODG as a valuable compound for future combination therapy research.

## Supplementary Materials

Supplementary Table The list of identified cortical proteins and DEPs in LODG and HODG via LC-MS/MS can be accessed via https://aperta.ulakbim.gov.tr/record/264976

Figure S1 KEGG pathway enrichment analysis of hub proteins. Three out of the top 5 hub proteins, which are indicated with a star, are enriched in the ‘RAS signaling’ KEGG pathway. Boxes with stars represent hub proteins as follows: Gβγ: GBB1 and GBB2, PAK: PAK2, Rho: RhoA.

Figure S2 KEGG pathway enrichment analysis of hub proteins. Three out of the top 5 hub proteins, which are indicated with a star, are enriched in the ‘Retrograde endocannabinoid signaling’ KEGG pathway. Boxes with stars represent hub proteins as follows: Gi/o: GBB1 and GBB2, Complex1: NDUFS6.

## Figures and Tables

**Table:** Number of identified and differentially expressed proteins (DEPs) in cortex, hippocampus, and cerebellum of GAL, LODG and HODG groups.

	Cortex		Hippocampus		Cerebellum	
	Identified	DEPs	Identified	DEPs	Identified	DEPs
GAL	2556	83	2275	0	2454	0
LODG	1597	0	1653	0	1334	43
HODG		1		0		54

## Data Availability

The datasets generated during the current study have been shared in the Supplementary Materials at the end. Further data can be requested from the corresponding author upon reasonable request.

## Footnotes

1
http://www.bioinformatics.com.cn/en

2
www.inkscape.org

## References

[b1-tjb-48-03-163] Athanasios A Charalampos V Vasileios T Ashraf G 2017 Protein-protein interaction (PPI) network: recent advances in drug discovery Current Drug Metabolism 18 5 10 10.2174/138920021801170119204832 28889796

[b2-tjb-48-03-163] Atlante A Valenti D Latina V Amadoro G 2022 Dysfunction of mitochondria in Alzheimer’s disease: ANT and VDAC interact with toxic proteins and aid to determine the fate of brain cells International Journal of Molecular Sciences 23 7722 10.3390/ijms23147722 35887070 PMC9316216

[b3-tjb-48-03-163] Baldaçara L Borgio JGF Moraes WA Lacerda ALT Montaño MBMM 2011 Cerebellar volume in patients with dementia Revista Brasileira de Psiquiatria 33 122 129 10.1590/S1516-44462011000200006 21829904

[b4-tjb-48-03-163] Bokoch GM 2003 Biology of the p21-activated kinases Annual Review of Biochemistry 72 743 781 10.1146/annurev.biochem.72.121801.161742 12676796

[b5-tjb-48-03-163] Bores GM Huger FP Petko W Mutlib AE Camacho F 1996 Pharmacological evaluation of novel Alzheimer’s disease therapeutics: acetylcholinesterase inhibitors related to galanthamine The Journal of Pharmacology and Experimental Therapeutics 277 728 738 8627552

[b6-tjb-48-03-163] Bouter Y Kacprowski T Weissmann R Dietrich K Borgers H 2014 Deciphering the molecular profile of plaques, memory decline and neuron loss in two mouse models for Alzheimer’s disease by deep sequencing Frontiers in Aging Neuroscience 6 10.3389/fnagi.2014.00075 PMC3997018 24795628

[b7-tjb-48-03-163] Cai R Wang Y Huang Z Zou Q Pu Y 2021 Role of RhoA/ROCK signaling in Alzheimer’s disease Behavioural Brain Research 414 113481 10.1016/j.bbr.2021.113481 34302876

[b8-tjb-48-03-163] Civiero L Greggio E 2018 PAKs in the brain: function and dysfunction Biochimica et Biophysica Acta - Molecular Basis of Disease 1864 444 453 10.1016/j.bbadis.2017.11.005 29129728

[b9-tjb-48-03-163] Cohn W Melnik M Huang C Teter B Chandra S 2021 Multi-omics analysis of microglial extracellular vesicles from human Alzheimer’s disease brain tissue reveals disease-associated signatures Frontiers in Pharmacology 12 10.3389/fphar.2021.766082 PMC8675946 34925024

[b10-tjb-48-03-163] D’Addario C Di Francesco A Trabace L Finazzi Agro A Cuomo V 2013 Endocannabinoid signaling in Alzheimer’s disease: current knowledge and future directions Journal of Biological Regulators and Homeostatic Agents 27 61 73 24813316

[b11-tjb-48-03-163] Dos Santos V Thomann PA Wüstenberg T Seidl U Essig M 2011 Morphological cerebral correlates of CERAD test performance in mild cognitive impairment and Alzheimer’s disease Journal of Alzheimer’s Disease 23 411 420 10.3233/JAD-2010-100156 21116054

[b12-tjb-48-03-163] Forner S Kawauchi S Balderrama-Gutierrez G Kramár EA Matheos DP 2021 Systematic phenotyping and characterization of the 5xFAD mouse model of Alzheimer’s disease Scientific Data 8 10.1038/s41597-021-01054-y PMC8519958 34654824

[b13-tjb-48-03-163] Fujita Y Yamashita T 2014 Axon growth inhibition by RhoA/ROCK in the central nervous system Frontiers in Neuroscience 8 10.3389/fnins.2014.00338 PMC4205828 25374504

[b14-tjb-48-03-163] Fukuda T Hiraide T Yamoto K Nakashima M Kawai T 2020 Exome reports A de novo GNB2 variant associated with global developmental delay, intellectual disability, and dysmorphic features European Journal of Medical Genetics 63 103804 10.1016/j.ejmg.2019.103804 31698099

[b15-tjb-48-03-163] Gauthier S Rosa-Neto P Morais JA Webster C 2021 World Alzheimer Report 2021: journey through the diagnosis of dementia Alzheimer’s Disease International 2022 30

[b16-tjb-48-03-163] Gellersen HM Guell X Sami S 2021 Differential vulnerability of the cerebellum in healthy ageing and Alzheimer’s disease Neuroimage: Clinical 30 10.1016/j.nicl.2021.102605 PMC7974323 33735787

[b17-tjb-48-03-163] Guo CC Tan R Hodges JR Hu X Sami S 2016 Network-selective vulnerability of the human cerebellum to Alzheimer’s disease and frontotemporal dementia Brain: A Journal of Neurology 139 1527 1538 10.1093/brain/aww003 26912642 PMC5839595

[b18-tjb-48-03-163] Gurel B Cansev M Koc C Ocalan B Cakir A 2019 Proteomics analysis of CA1 region of the hippocampus in pre-, progression and pathological stages in a mouse model of the Alzheimer’s disease Current Alzheimer Research 16 613 621 10.2174/1567205016666190730155926 31362689

[b19-tjb-48-03-163] Hemati P Revah-Politi A Bassan H Petrovski S Bilancia CG 2018 Refining the phenotype associated with GNB1 mutations: clinical data on 18 newly identified patients and review of the literature American Journal of Medical Genetics Part A 176 2259 2275 10.1002/ajmg.a.40472 30194818

[b20-tjb-48-03-163] Huesa G Baltrons MA Gómez-Ramos P Morán A García A 2010 Altered distribution of RhoA in Alzheimer’s disease and AβPP overexpressing mice Journal of Alzheimer’s disease 19 37 56 10.3233/JAD-2010-1203 20061625

[b21-tjb-48-03-163] Katona I 2006 Molecular composition of the endocannabinoid system at glutamatergic synapses Journal of Neuroscience 26 5628 5637 10.1523/JNEUROSCI.0309-06.2006 16723519 PMC1698282

[b22-tjb-48-03-163] Kiris I Basar MK Sahin B Gurel B Coskun J 2021 Evaluation of the therapeutic effect of lycoramine on Alzheimer’s disease in mouse model Current Medicinal Chemistry 28 3449 3473 10.2174/0929867327999201116193126 33200692

[b23-tjb-48-03-163] Kiris I Kukula-Koch W Karayel-Basar M Gurel B Coskun J 2023 Proteomic alterations in the cerebellum and hippocampus in an Alzheimer’s disease mouse model: alleviating effect of palmatine Biomedicine & Pharmacotherapy 158 114111 10.1016/j.biopha.2022.114111 36502756

[b24-tjb-48-03-163] Kiris I Skalicka-Wozniak K Basar MK Sahin B Gurel B 2022 Molecular effects of pteryxin and scopoletin in the 5xFAD Alzheimer’s disease mouse model Current Medicinal Chemistry 29 2937 2950 10.2174/0929867328666210827152914 34455957

[b25-tjb-48-03-163] Lam M Trampush JW Yu J Knowles E Davies G 2017 Large-scale cognitive GWAS meta-analysis reveals tissue-specific neural expression and potential nootropic drug targets Cell Reports 21 2597 2613 10.1016/j.celrep.2017.11.028 29186694 PMC5789458

[b26-tjb-48-03-163] Lansdon LA Fleming EA Del Viso F Sullivan BR Saunders CJ 2021 Second patient with GNB2-related neurodevelopmental disease: further evidence for a gene-disease association European Journal of Medical Genetics 64 104243 10.1016/j.ejmg.2021.104243 33971351

[b27-tjb-48-03-163] Liu Y Tan Y Zhang Z Yi M Zhu L 2024 The interaction between ageing and Alzheimer’s disease: insights from the hallmarks of ageing Translational Neurodegeneration 13 7 10.1186/s40035-024-00397-x 38254235 PMC10804662

[b28-tjb-48-03-163] López S Bastida J Viladomat F Codina C 2002 Acetylcholinesterase inhibitory activity of some Amaryllidaceae alkaloids and *Narcissus* extracts Life Sciences 71 2521 2529 10.1016/S0024-3205(02)02034-9 12270757

[b29-tjb-48-03-163] Ludányi A Hu SSJ Yamazaki M Tanimura A Piomelli D 2011 Complementary synaptic distribution of enzymes responsible for synthesis and inactivation of the endocannabinoid 2-arachidonoylglycerol in the human hippocampus Neuroscience 174 50 63 10.1016/j.neuroscience.2010.10.062 21035522 PMC3678284

[b30-tjb-48-03-163] Marlin JW Chang YE Ober M Handy A Xu W 2011 Functional PAK-2 knockout and replacement with a caspase cleavage-deficient mutant in mice reveals differential requirements of full-length PAK-2 and caspase-activated PAK-2p34 Mammalian Genome 22 306 317 10.1007/s00335-011-9326-6 21499899

[b31-tjb-48-03-163] Martins-De-Souza D Gattaz WF Schmitt A Rewerts C Marangoni S 2009 Alterations in oligodendrocyte proteins, calcium homeostasis and new potential markers in schizophrenia anterior temporal lobe are revealed by shotgun proteome analysis Journal of Neural Transmission 116 275 289 10.1007/s00702-008-0156-y 19034380

[b32-tjb-48-03-163] Martins-de-Souza D Schmitt A Röder R Lebar M Schneider-Axmann T 2010 Sex-specific proteome differences in the anterior cingulate cortex of schizophrenia Journal of Psychiatric Research 44 989 991 10.1016/j.jpsychires.2010.03.003 20381070

[b33-tjb-48-03-163] Mroczek T 2009 Highly efficient, selective and sensitive molecular screening of acetylcholinesterase inhibitors of natural origin by solid-phase extraction-liquid chromatography/electrospray ionisation-octopole-orthogonal acceleration time-of-flight-mass spectrometry and novel thin-layer chromatography-based bioautography Journal of Chromatography A 1216 2519 2528 10.1016/j.chroma.2009.01.061 19203760

[b34-tjb-48-03-163] Mroczek T 2016 Qualitative and quantitative two-dimensional thin-layer chromatography/high performance liquid chromatography/diode-array/electrospray-ionization-time-of-flight mass spectrometry of cholinesterase inhibitors Journal of Pharmaceutical Biomedical Analysis 129 155 162 10.1016/j.jpba.2016.06.048 27424196

[b35-tjb-48-03-163] Mroczek T Dymek A Widelski J Wojtanowski KK 2020 The bioassay-guided fractionation and identification of potent acetylcholinesterase inhibitors from *Narcissus c.v.*“Hawera” using optimized vacuum liquid chromatography, high resolution mass spectrometry and bioautography Metabolites 10 1 16 10.3390/metabo10100395 PMC7599570 33020380

[b36-tjb-48-03-163] Nguyen TVV Galvan V Huang W Banwait S Tang H 2008 Signal transduction in Alzheimer disease: p21-activated kinase signaling requires C-terminal cleavage of APP at Asp664 Journal of Neurochemistry 104 1065 1080 10.1111/j.1471-4159.2007.05031.x 17986220 PMC2553705

[b37-tjb-48-03-163] Oakley H Cole SL Logan S Maus E Shao P 2006 Intraneuronal β-amyloid aggregates, neurodegeneration, and neuron loss in transgenic mice with five familial Alzheimer’s disease mutations: potential factors in amyloid plaque formation Journal of Neuroscience 26 10129 10140 10.1523/JNEUROSCI.1202-06.2006 17021169 PMC6674618

[b38-tjb-48-03-163] Pang X Zhao Y Wang J Zhou Q Xu L 2017 The bioinformatic analysis of the dysregulated genes and microRNAs in entorhinal cortex, hippocampus, and blood for Alzheimer’s disease Biomed Research International 2017 10.1155/2017/9084507 PMC5735586 29359159

[b39-tjb-48-03-163] Peng Y Chang X Lang M 2021 Iron homeostasis disorder and Alzheimer’s disease International Journal of Molecular Sciences 22 12442 10.3390/ijms222212442 34830326 PMC8622469

[b40-tjb-48-03-163] Petratos S Li QX George AJ Hou X Kerr ML 2008 The β-amyloid protein of Alzheimer’s disease increases neuronal CRMP-2 phosphorylation by a Rho-GTP mechanism Brain 131 90 108 10.1093/brain/awm260 18000012

[b41-tjb-48-03-163] Petrovski S Küry S Myers CT Anyane-Yeboa K Cogné B 2016 Germline de novo mutations in GNB1 Cause severe neurodevelopmental disability, hypotonia, and seizures American Journal of Human Genetics 98 1001 1010 10.1016/j.ajhg.2016.03.011 27108799 PMC4863562

[b42-tjb-48-03-163] Picard H Amado I Mouchet-Mages S Olié JP Krebs MO 2008 The role of the cerebellum in schizophrenia: an update of clinical, cognitive, and functional evidences Schizophrenia Bulletin 34 155 72 10.1093/schbul/sbm049 17562694 PMC2632376

[b43-tjb-48-03-163] Qu L Pan C He SM Lang B Gao GD 2019 The Ras superfamily of small GTPases in non-neoplastic cerebral diseases Frontiers in Molecular Neuroscience 12 10.3389/fnmol.2019.00121 PMC6555388 31213978

[b44-tjb-48-03-163] Sánchez C de Ceballos ML Gomez del Pulgar T Rueda D Corbacho C 2001 Inhibition of glioma growth in vivo by selective activation of the CB(2) cannabinoid receptor Cancer Research 61 5784 9 11479216

[b45-tjb-48-03-163] Santini E Huynh TN Longo F Koo SY Mojica E 2017 Reducing eIF4E-eIF4G interactions restores the balance between protein synthesis and actin dynamics in fragile X syndrome model mice Science Signalling 10 10.1126/scisignal.aan0665 PMC5858943 29114037

[b46-tjb-48-03-163] Sastre AA Montoro ML Gálvez-Martín P Lacerda HM Lucia A 2020 Small GTPases of the Ras and rho families switch on/off signaling pathways in neurodegenerative diseases International Journal of Molecular Science 21 1 23 10.3390/ijms21176312 PMC7504559 32878220

[b47-tjb-48-03-163] Savage JE Jansen PR Stringer S Watanabe K Bryois J 2018 Genome-wide association meta-analysis in 269, 867 individuals identifies new genetic and functional links to intelligence Nature Genetics 50 912 919 10.1038/s41588-018-0152-6 29942086 PMC6411041

[b48-tjb-48-03-163] Schmidt SI Blaabjerg M Freude K Meyer M 2022 RhoA signaling in neurodegenerative diseases Cells 11 1520 10.3390/cells11091520 35563826 PMC9103838

[b49-tjb-48-03-163] Shin EY Shim ES Lee CS Kim HK Kim EG 2009 Phosphorylation of RhoGDI1 by p21-activated kinase 2 mediates basic fibroblast growth factor-stimulated neurite outgrowth in PC12 cells Biochemical and Biophysical Research Communications 379 384 389 10.1016/j.bbrc.2008.12.066 19103160

[b50-tjb-48-03-163] Stankiewicz TR Linseman DA 2014 Rho family GTPases: key players in neuronal development, neuronal survival, and neurodegeneration Frontiers in Cellular Neuroscience 8 10.3389/fncel.2014.00314 PMC4187614 25339865

[b51-tjb-48-03-163] Syrovatkina V Alegre KO Dey R Huang XY 2016 Regulation, signaling, and physiological functions of G-proteins Journal of Molecular Biology 428 3850 3868 10.1016/j.jmb.2016.08.002 27515397 PMC5023507

[b52-tjb-48-03-163] Tanvir Kabir M Sahab Uddin M Al Mamun A Jeandet P Aleya L 2020 Combination drug therapy for the management of Alzheimer’s disease International Journal of Molecular Sciences 21 10.3390/ijms21093272 PMC7246721 32380758

[b53-tjb-48-03-163] Uddin MS Al Mamun A Kabir MT Ashraf GM Bin-Jumah MN 2021 Multi-target drug candidates for multifactorial Alzheimer’s disease: AChE and NMDAR as molecular targets Molecular Neurobiology 58 281 303 10.1007/s12035-020-02116-9 32935230

[b54-tjb-48-03-163] Wang Y Zeng C Li J Zhou Z Ju X 2018 PAK2 Haploinsufficiency results in synaptic cytoskeleton impairment and autism-related behavior Cell Reports 24 2029 2041 10.1016/j.celrep.2018.07.061 30134165

[b55-tjb-48-03-163] Wassink TH Andreasen NC Nopoulos P Flaum M 1999 Cerebellar morphology as a predictor of symptom and psychosocial outcome in schizophrenia Biological Psychiatry 45 41 8 10.1016/s0006-3223(98)00175-9 9894574

[b56-tjb-48-03-163] Yamada K Nabeshima T 2000 Animal models of Alzheimer’s disease and evaluation of anti-dementia drugs Pharmacology & Therapeutics 88 93 113 10.1016/S0163-7258(00)00081-4 11150591

[b57-tjb-48-03-163] Zhang K Wang Y Fan T Zeng C Sun ZS 2022 The p21-activated kinases in neural cytoskeletal remodeling and related neurological disorders Protein & Cell 13 6 25 10.1007/s13238-020-00812-9 33306168 PMC8776968

