# Supplementary figures and images for: O-demethyl galantamine alters protein expression in cerebellum of 5xFAD mice
Source: Turk J Biol. 2024 May 28;48(3):163–73. doi: 10.55730/1300-0152.2692 (PMC11265889; doi:10.55730/1300-0152.2692)

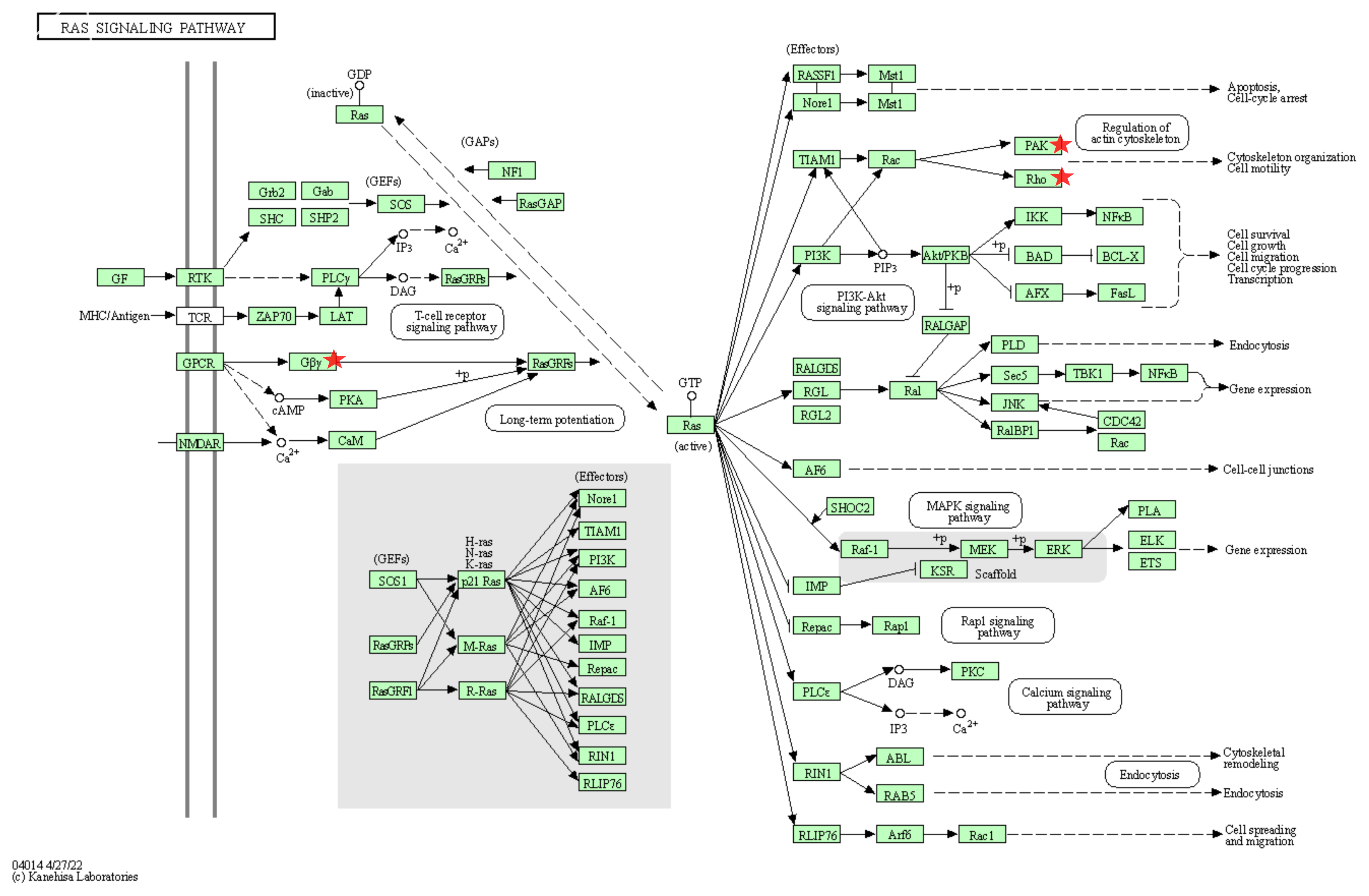

Supplement: Figure S1 — KEGG pathway enrichment analysis of hub proteins. Three out of the top 5 hub proteins, which are indicated with a star, are enriched in the ‘RAS signaling’ KEGG pathway. Boxes with stars represent hub proteins as follows: Gβγ: GBB1 and GBB2, PAK: PAK2, Rho: RhoA. [file tjb-48-03-163s1.tif]

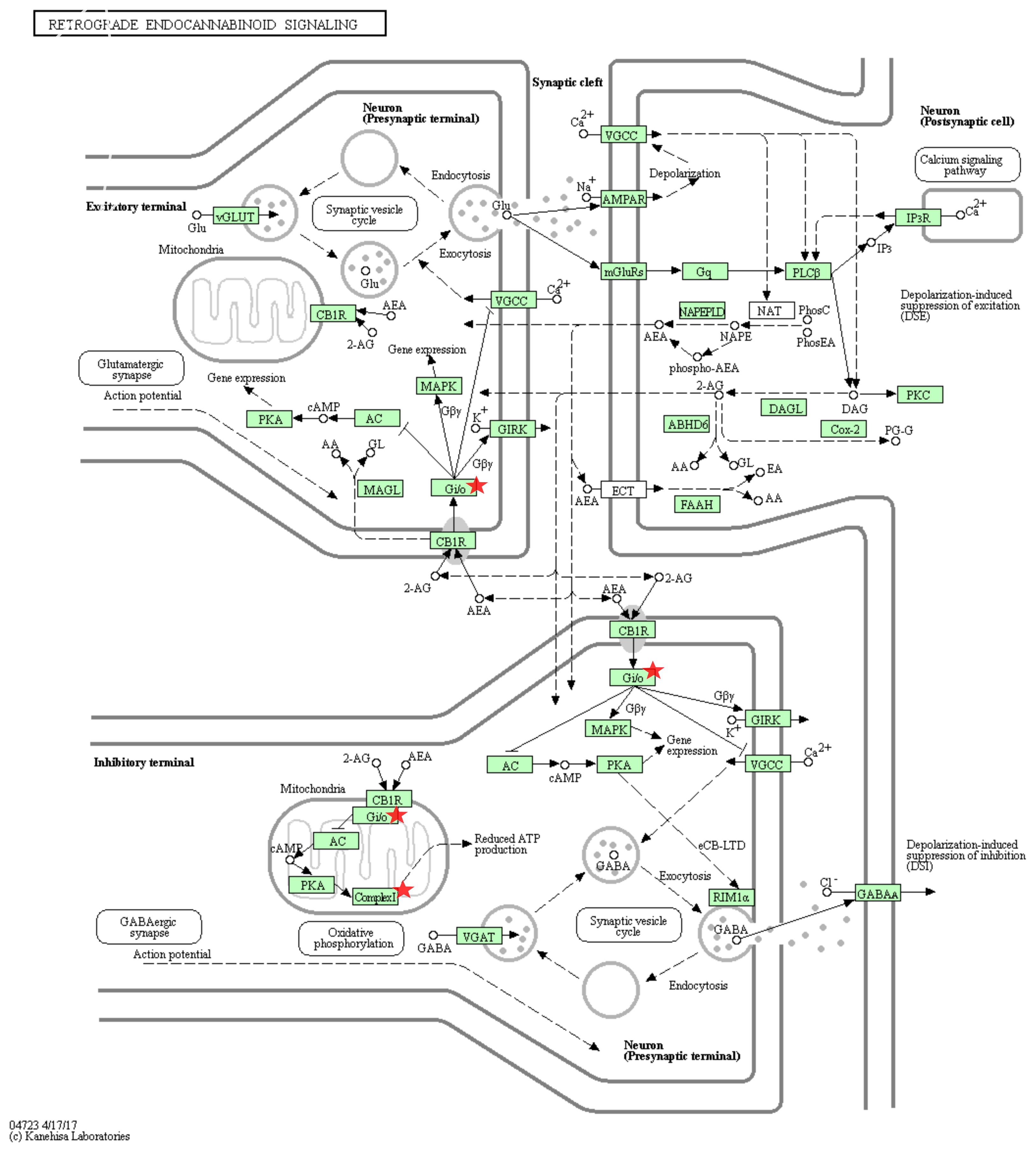

Supplement: Figure S2 — KEGG pathway enrichment analysis of hub proteins. Three out of the top 5 hub proteins, which are indicated with a star, are enriched in the ‘Retrograde endocannabinoid signaling’ KEGG pathway. Boxes with stars represent hub proteins as follows: Gi/o: GBB1 and GBB2, Complex1: NDUFS6. [file tjb-48-03-163s2.tif]
